# Three sympatric karyomorphs in the fish *Astyanax fasciatus* (Teleostei, Characidae) do not seem to hybridize in natural populations

**DOI:** 10.3897/CompCytogen.v6i1.2151

**Published:** 2012-01-24

**Authors:** Maressa Ferreira-Neto, Roberto Ferreira Artoni, Marcelo Ricardo Vicari, Orlando Moreira-Filho, Juan Pedro Martínez Camacho, Mohammed Bakkali, Claudio de Oliveira, Fausto Foresti

**Affiliations:** 1 Departmento de Morfologia, Instituto de Biociências, Universidade Estadual Paulista, Distrito de Rubião Jr, Botucatu, São Paulo, Brazil; 2 Departamento de Biologia Estrutural, Molecular e Genética, Universidade Estadual de Ponta Grossa, Ponta Grossa, Paraná, Brazil; 3 Programa de Pós-Graduação em Genética e Evolução, Universidade Federal de São Carlos, São Carlos, São Paulo, Brazil; 4 Departamento de Genética, Universidad de Granada, 18071 Granada, Spain

**Keywords:** fish cytogenetics, chromosome banding, rDNA, sympatric differentiation, B chromosome

## Abstract

Ninety individuals of the characid fish *Astyanax fasciatus* (Cuvier, 1819) were collected at Água da Madalena stream (Botucatu, São Paulo, Brazil) and analyzed for diploid chromosome number 2n and karyotype composition as well as for the chromosomal location of the 5S and 18S ribosomal DNA (rDNA). Whereas no chromosome differences were associated with sex, three different karyomorphs with diploid chromosome numbers 2n=46, 2n=48 and 2n=50 were found. No intermediate 2n numbers were discovered. The 2n=50 karyomorph showed some differences in 18S rDNA location compared to the two other karyomorphs. Finally, all specimens with the 2n=46 karyomorph showed the presence of a partly heterochromatic macro supernumerary chromosome, which was absent in all individuals with the two other karyomorphs. All these results suggest that indviduals of the three different karyomorphs are not likely to hybridize in the examined populations. Our findings strongly suggest the presence of three separate species (*sensu* biological species concept) easily diagnosed on the basis of differences in the diploid chromosome numbers and other chromosomal markers.

## Introduction

The genus *Astyanax* (Baird et Girard, 1854) is one of the most diversified among Neotropical characid fishes. Indeed, this genus shows an extensive morphological diversification and a highly complex taxonomy. Moreover, a number of species actually appear to be “complexes of species” with low morphological differentiation but high variation at other levels, e.g. chromosome number and morphology (Morelli et al. 1983). The first *Astyanax* “species complex” was suggested by Moreira-Filho and Bertollo in *Astyanax scabripinnis* Jenyns, 1842 (Moreira-Filho and Bertollo 1991). Other cases were reported in the Neotropical fish *Corydoras aeneus* Gill, 1858 (Turner et al. 1992), *Hoplias malabaricus*, Bloch 1794 (Bertollo et al. 1997), and *Gymnotus carapo* Linnaeus, 1758 (Milhomem et al. 2008).

**Figure 1.**
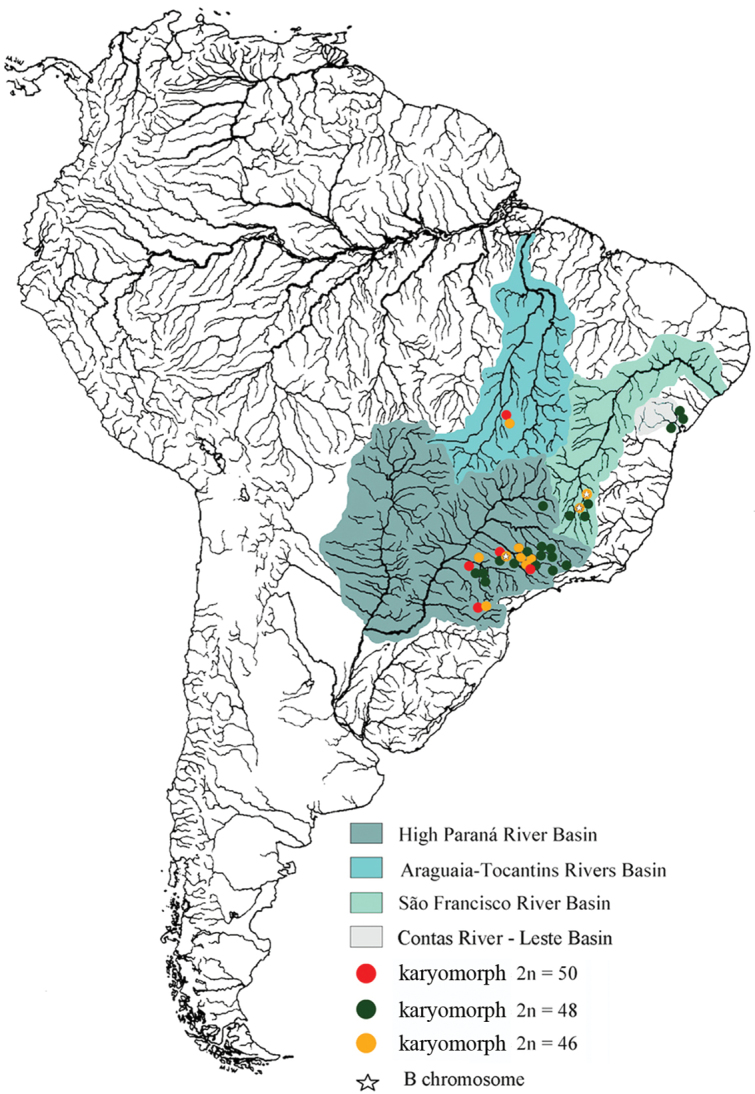
Map of South America highlighting the hydrographic basin of the Paraná, Araguaia-Tocantins, Contas and São Francisco Rivers. The natural distribution of *Astyanax fasciatus* and the presence of B chromosomes are represented according to the legends.

*Astyanax fasciatus* is another species that seems to form a “species complex” since the available information points towards the existence of several karyomorphs with the diploid chromosome numbers 2n=45, 2n=46, 2n=47, 2n=48 and 2n=50 (Table 1). The karyotype most frequently cited in the literature is 2n=48 (Table 1), but the known geographical range is actually larger for the karyomorphs 2n=50 and 2n=46 (Figure 1).

**Table 1.** Diploid numbers (2n) recorded in populations of *Astyanax fasciatus* complex.

**Species as originally reported**	**River/basin**	**2n**	**B chrom.**	**Reference**
*Astyanax fasciatus*	Mogi Guaçu river/Paraná	45		14
*Astyanax fasciatus*	Mogi Guaçu river/Paraná	46		2,4,9,10,11,18
*Astyanax fasciatus*	Mogi Guaçu river/Paraná	47		14,11
*Astyanax fasciatus*	Mogi Guaçu river/Paraná	48		11,18
*Astyanax fasciatus*	Piracicaba river/Paraná	48		7
*Astyanax fasciatus*	Piracicaba river/Paraná	46		23
*Astyanax fasciatus*	Tietê river/Paraná	46		5
*Astyanax fasciatus*	Riacho Águas da Madalena/Paraná	50		23
*Astyanax fasciatus*	Águas da Madalena stream/Paraná	48		23
*Astyanax fasciatus*	Águas da Madalena stream/Paraná	46	+	23
*Astyanax fasciatus*	Paranapanema river/Paraná	46		6
*Astyanax fasciatus*	Paranapanema river/Paraná	50		13
*Astyanax fasciatus*	Paraíba river/Paraná	48		3,5,9
*Astyanax fasciatus*	Paiol Grande river/Paraná	48		9,17
*Astyanax fasciatus*	Barra funda river/Paraná	46		9
*Astyanax fasciatus*	Passa Cinco river/Paraná	46		9,11
*Astyanax fasciatus*	Sapucaí river/Paraná	48		12
*Astyanax fasciatus*	Araguari river/Paraná	46		16
*Astyanax* cf. *fasciatus*	Juquiá river/Paraná	48		2
*Astyanax fasciatus*	Meia Ponte river/Araguaia	46		1
*Astyanax* prope *fasciatus*	Córrego Fundo stream/Araguaia	50		8
*Astyanax fasciatus*	Araras river/Paraná	48		22
*Astyanax fasciatus*	Patos river/Paraná	48		22
*Astyanax fasciatus*	Três Bueiros river/Paraná	48		22
*Astyanax fasciatus*	Almas river/Paraná	48		22
*Astyanax* prope *fasciatus*	Tibagi river/Paraná	48		19
*Astyanax* prope *fasciatus*	Tibagi river/Paraná	49		19
*Astyanax* prope *fasciatus*	Tibagi river/Paraná	50		19
*Astyanax fasciatus*	São Francisco river/São Francisco	48		7,9,17,22
*Astyanax fasciatus*	São Francisco river/São Francisco	46	+	7
*Astyanax fasciatus*	Três Marias/São Franscisco	46	+	15
*Astyanax fasciatus*	Contas river/Leste	48		21
*Astyanax fasciatus*	Mineiro do Costa stream/Leste	48		21
*Astyanax fasciatus*	Preto do Costa river/Leste	48		21

References: 1. Jim and Toledo (1975); 2. Morelli et al. (1983); 3. Moreira-Filho and Bertollo (1986); 4. Paganelli (1990); 5. Justi et al. (1990); 6. Reganham and Giuliano-Caetano (1990); 7. Justi (1993); 8. Centofante and Vênere (1995); 9. Heras and Moreira-Filho (1996); 10. Daniel-Silva (1996); 11. Heras and Moreira-Filho (1997); 12. Swerts et al. (1998); 13. Vale and Martins-Santos (1999); 14. Daniel-Silva and Almeida-Toledo (2001); 15. Moreira-Filho et al. (2001); 16. Torres-Mariano and Morelli (2006); 17. Abel et al. (2006); 18. Pazza et al. (2006); 19. Artoni et al. (2006); 21. Medrado et al. (2008); 22. Peres et al. (2009); 23. Ferreira-Neto et al. (present study).

B chromosomes are supernumerary elements previously reported in the karyotypes of several *Astyanax* species (Moreira-Filho et al. 2004). In *Astyanax fasciatus*, B chromosomes have been reported only for the 2n=46 karyomorph in a population at the Sao Francisco River basin (Moreira-Filho et al. 2001).

In this study we analyzed the 2n=46, 2n=48 and 2n=50 *Astyanax fasciatus* karyomorphs by examining a high number of individuals living in sympatry. The absence of intermediate chromosome numbers, the restriction of B chromosomes to the 2n=46 karyomorph and the absence of heteromorphism for the 18S rDNA sites (which differ among some karyomorphs) strongly suggest that the abovementioned karyomorphs are not likely to hybridize and are thus three separate species (*sensu* biological species concept).

## Material and methods

Ninety *Astyanax fasciatus* specimens (56 females and 34 males) were collected at a 3.000 m^2^ area of the Água da Madalena stream, Botucatu, SP, Brazil (22°59'23"S, 48°25'31"W). The specimens were anaesthetized in benzocaine (1%) and, after collecting tissue samples for chromosome analysis, fixed in 10% formol and preserved in 70% alcohol. After identification, the specimens were deposited in the Museum of Biology and Laboratory of Fish Genetics, UNESP, Botucatu, São Paulo, Brazil. Chromosome preparations were obtained from anterior kidney cells and used for the conventional air drying technique (Foresti et al. 1981). The chromosomal location of active nucleolus organizer regions (NORs) was detected using the silver nitrate staining technique (Howell and Black 1980). Mapping of the ribosomal DNA (rDNA) was performed by fluorescent *in situ* hybridization (FISH) according to Pinkel et al. (1986). The 18S and 5S rDNA probes were obtained from the fish *Prochilodus argenteus* Spix and Agassiz, 1829 (Hatanaka and Galetti Jr. 2004) and *Leporinus elongatus* Valenciennes, 1850 (Martins and Galetti Jr. 1999), respectively. The 5SS probe was labeled with biotin 14-dATP by nick translation following manufacturer’s instructions (Bionick Labelling System - Invitrogen). Hybridization was detected with avidin-FITC and the signals were amplified with biotinylated anti-avidin. The 18S probe was labeled with digoxigenin 11-dUTP (Roche Applied Sciences) by PCR (Polymerase Chain Reaction) and hybridization signals were detected using anti-digoxigenin-rhodamine.

Metaphase chromosomes were counterstained with DAPI and analyzed under optical light microscope (Olympus BX61). Images were captured using the Image-Pro Plus 6.0 software (Media Cybernetics). To build karyograms, chromosome morphology was determined according to the arm ratio limits established by Levan et al. (1964), and chromosomes were classified as metacentric (m), submetacentric (sm), subtelocentric (st) and acrocentric (a), and were arranged in order of decreasing size.

## Results

**Figure 2.**
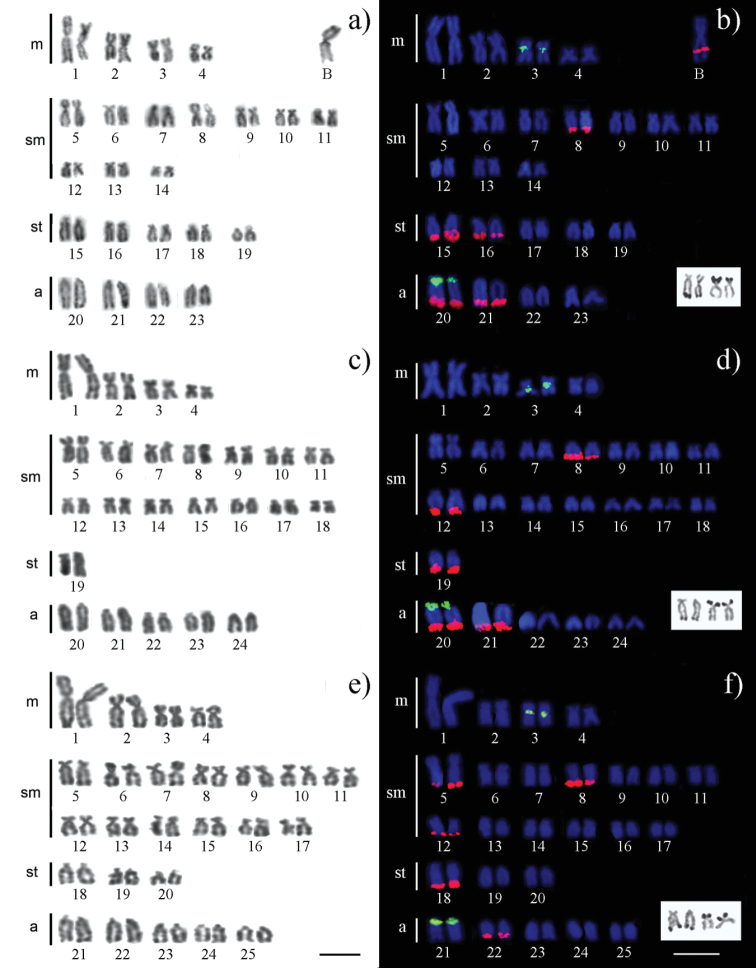
Karyotypes of *Astyanax fasciatus* (2n=46, 48 and 50 chromosomes) deduced after conventional Giemsa staining (a, c, e) and double FISH using 5S (green) and 18S rDNA (red) probes. The chromosomes bearing the Ag-NORs are boxed in b, d and f. Bar = 10 μm.

The cytogenetic analysis of the 90 specimens of *Astyanax fasciatus* revealed the presence of three different karyomorphs in the sample, showing 2n=46, 2n=48 and 2n=50 chromosomes (Table 2 and Fig. 2a, c and e, respectively), and with fundamental numbers (NF= number of chromosome arms) equal to 84, 86 and 90, respectively. The anatomical sex of the fish, determined by visual examination of the gonads, was not associated with the karyotypic differences, so that males and females were found with all the three cytotypes described here. Interestingly, all the specimens with the 2n=46 karyomorph showed the presence of one mitotically stable macro B chromosome (Fig. 2a, b), whereas the specimens of the 2n=48 and 2n=50 karyomorphs lacked it. No individual with intermediate odd chromosome numbers was found.

**Table 2.** Cytogenetic studies in *Astyanax fasciatus* karyotype composition and location of the 5S and 18S rDNA in the chromosomes of the individuals analyzed.

**Cytotypes 2n / NF**	**Number of specimens**	**Chromosome**	**rDNA 18S/5S**	**Number of B Chromosomes**		**NOR**
	**Female Male**	**Formulae**	**location (pairs)**	**females**	**males**	**(pairs)**
*2n = 46, NF=84*	12 10	5m+8sm+6st+4a	8,15,16,20,21, B/3,20	1	1	9,10
*2n = 48, NF=86*	10 9	5m+8sm+6st+5a	8,12,19,20,21/3,20	0	0	9,10
*2n = 50, NF=84*	41 32	5m+8sm+4st+8a	5,8,12,18,22/3,21	0	0	9,10

Physical mapping of the rDNA showed remarkable conservation of the 5S rDNA sites, which were present in two chromosome pairs, i.e. Nos. 3 and 20 (21 in the 2n=50 karyomorph) (Fig. 2b, d, f).

The silver staining technique revealed the presence, in all cells, of Ag-NORs on two sm chromosome pairs of the three karyomorphs. One Ag-NOR was located at the distal region of the p arm and the other at the q arm (Figs 2b, d, f, details). FISH analysis, however, showed the presence of 18S rDNA at the distal regions of five chromosome pairs, but no FISH signal was observed on the short arm of any chromosome (Fig. 2b, d, f). This points towards the presence of a small number of 18S rRNA genes in the short arm of the sm chromosome.

Double FISH showed the presence of a chromosome pair, No. 20 in the 2n=46 and 2n=48 karyomorph, carrying both 5S (proximal) and 18S (distal) rDNA, but no chromosome carried both kinds of rDNA in the 2n=50 karyomorph (Fig. 1f). All six specimens analyzed by FISH were homomorphic for the chromosome No. 20 which carries both rDNA types. Remarkably, FISH mapping showed the presence of an interstitial cluster of 18S rDNA in the long arm of the B chromosome found in the 2n=46 karyomorph. Nevertheless, this rDNA cluster was never detected by silver staining.

## Discussion

Sympatry and syntopy for several cytotypes have been reported in *Astyanax* species, such as *Astyanax scabripinnis* (Souza and Moreira-Filho 1995) and *Astyanax fasciatus* (Pazza et al. 2006, 2008). A review of all the published data on *Astyanax fasciatus* (summarized in Table 1 and Figure 1) indicates that the three karyomorphs reported in this work show broad geographical distribution, and sympatry can be observed for at least two different karyomorphs in four river basins, i.e. High Paraná, Araguaia-Tocantins, Contas and São Francisco. In addition, B chromosomes have been previously reported in two other populations from the São Francisco River basin although, in that case, the number of B chromosomes showed variation among individuals (Justi 1993, Heras and Moreira-Filho 1997, Moreira-Filho et al. 2001). One evidence in support of hybridization events between karyomorphs was reported by Artoni et al. (2006), who found an individual with 49 chromosomes which might be a hybrid between the 48 and the 50 cytotypes in the Tibagi River (High Paraná River Basin, Ponta Grossa, Paraná, Brazil). The other evidence supporting hybridization between karyomorphs was reported by Pazza et al. (2006, 2008) who identified karyomorphs with 2n=45, 46, 47 and 48 chromosomes in individuals caught along the Mogi-Guaçu river (High Paraná River Basin, Cachoeira de Emas, São Paulo, Brazil). In this last case, the 2n=47 chromosomes karyomorph could have resulted from hybridization between the 46 and 48 cytotypes, whereas the presence of three individuals with 45 chromosomes could suggest the possible existence of a karyomorph with 2n=44 in that river.

Our present analysis of a large sample of individuals caught in the same stream shows syntopic occurrence of three *Astyanax fasciatus* karyomorphs with discrete chromosome numbers 2n=46, 2n=48 and 2n=50. The absence of intermediate chromosome numbers, the presence of B chromosomes in only one of these karyomorphs (2n=46), and the absence of apparent heteromorphism for the chromosome 20, suggests that the three karyomorphs do not hybridize in this stream. In such case, the data point out towards the possibility that these three karyomorphs actually correspond to three cryptic species, thus supporting the hypothesis that *Astyanax fasciatus* is in fact a species assemblage, i.e. several species were included under the nominal name of *Astyanax fasciatus* (Artoni et al. 2006).

The B chromosome found in *Astyanax fasciatus* is large and metacentric. Interestingly, both characteristics seem common to all the B chromosomes described *Astyanax* species including *Astyanax scabripinnis* (Salvador and Moreira-Filho 1992, Maistro et al. 1992, Souza and Moreira-Filho 1995, Vicente et al. 1996, Mizoguchi and Martins-Santos 1997, Vicari et al. 2010, 2011), *Astyanax eigenmanniorum* Cope, 1894 (Fauaz et al. 1994), *Astyanax schubarti* Britski, 1964 (Moreira-Filho 2001), *Astyanax fasciatus*, (Moreira-Filho 2001) and *Astyanax bockmanni*, Vari & Castro 2007 (Daniel personal communication). Such morphological similarity would support the hypothesis of a common origin of the *Astyanax* B chromosomes (Moreira-Filho et al. 2001). Nonetheless we show here, for the first time, the presence of rDNA interstitially located in the p arm of the B chromosome in *Astyanax fasciatus* individuals. The apparent absence of rDNA in the q arm may seem incompatible with the isochromosome origin previously suggested for some of these Bs, e.g. in *Astyanax scabripinnis* (Mestriner et al. 2000). However, the presence of a small, bellow FISH sensitivity, amount of rRNA genes is also possible. A genus-wise investigation of the presence and distribution of the rDNA in *Astyanax* species may be very useful for inferring the origin of the B chromosome. To this end, a comparison of rDNA sequences among the A and B chromosomes of several species would be very informative.

Since silver staining of metaphasic chromosomes reveals only those NORs that were active in the previous interphase (Hsu et al. 1975), it seems that the 18S rDNA contained in the B chromosome is usually inactive. Inactivity of the rDNA seems thus a widespread and general feature of the B chromosomes as it was previously reported in several phylogenetically distant species including the grasshopper *Eyprepocnemis plorans* Charpentier, 1825 (Cabrero et al. 1997, Bakkali et al. 2001), the black rat *Rattus rattus* Linnaeus, 1758 (Stitou et al. 2000) and the fish *Haplochromis obliquidens* Hilgendorf, 1888(Poletto et al. 2010). Nonetheless, active rRNA genes have ocasionally been reported in some B chromosomes of species like the grasshopper *Eyprepocnemis plorans* (Teruel et al. 2007, 2009) and the rodents *Akodon montensis* Thomas, 1913and *Oryzomys angouya* Fischer, 1814 (Silva et al. 2004).

The higher number of rDNA clusters (10) than silver stained NORs (4) suggests the inactivity of some rDNAs in most cells. It would therefore be interesting to ascertain whether this phenomenon is facultative or constitutive. Silver nitra te may also bind to other proteins present in the nuclei, implying that some chromosome structures visualized by silver nitrate may not correspond to ribosomal genes (Dobigny et al. 2002). The detection of a higher number of ribosomal genes using FISH against 18S or 28S sequences than using silver nitrate staining is a common result having been reported in *Astyanax scabripinnis* (Ferro et al. 2001), *Salmo truta* (Pendás et al. 1993), *Colossoma macropomun* Cuvier, 1816, *Piaractus brachypomus* Cuvier, 1818 and their interspecific hybrids (Nirchio et al. 2003), *Hyphessobrycon anisitsi* Eigenmann, 1907 (Centofante et al. 2003), *Prochilodus lineatus* Valenciennes, 1836 (Jesus and Moreira-Filho 2003) and *Lebias fasciata* Valenciennes, 1821 (Tigano et al. 2004). Therefore, the chromosome pair that actually has the nucleolus organizer region is probably the first submetacentric pair (the one that shows both FISH and silver nitrate staining signals).

The presence of one B chromosome in all 22 individuals from the 2n=46 karyomorph is intriguing, since it departs from the usual interindividual variation which characterizes B chromosomes (Camacho 2005). On the other hand, Moreira-Filho et al. (2001) reported the presence of B chromosomes showing interindividual variation in number in 10% of the sample population analyzed at the Sao Francisco River basin. This suggests that the B chromosomes in *Astyanax fasciatus* from the Água da Madalena stream (this report) might be in a state of stabilization. With complete elimination through one sex and complete drive through the other, the population dynamics of *Astyanax* B chromosome resembles the case of germ-line restricted chromosomes in the zebra finch (Itoh et al. 2009). The mechanism behind B chromosome stabilization in populations of the Água da Madalena stream appears therefore to be rather complex and its elucidation requires further population dynamics and chromosome transmission studies.
